# The safety and clinical impact of ultra-low-dose FDG-PET imaging in pregnancy-associated breast cancer: the experience of a major tertiary oncology referral centre in the UK and suggested imaging protocol

**DOI:** 10.1097/MNM.0000000000001997

**Published:** 2025-06-04

**Authors:** Karen Desouza, Petros Fessas, Emma Spurrell, Elisavet Papadimitraki, Fharat Raja, Rebecca Roylance, Diego Ottaviani, Joanna Franks, Dibendu Betal, Mahwash Babar, Irene Athanasiou, Massimilano Cariati, Neill Patani, Sirwan Hadad, Yusuf Kayani, Darren Walls, Catherine Scott, Marilena Rega, Mubarik Arshad, Jamshed Bomanji, Stefan Vöö

**Affiliations:** a Cancer Division, Department of Oncology, University College London Hospitals NHS Foundation Trust (UCLH); b Surgery and Cancer Integrated Clinical Service Unit, Whittington Health NHS Trust; c Department of Oncoplastic Breast Surgery, Women’s Health Division, UCLH; d Institute of Women’s Health, University College London; e Institute of Nuclear Medicine, UCLH, London, UK

**Keywords:** breast cancer, PET, pregnancy, pregnancy-associated cancer, pregnant

## Abstract

**Background:**

Pregnancy-associated breast cancer (PABC) is a complex condition affecting 1 in 3000 pregnancies worldwide. While clinical management has improved, the optimal staging approach for PABC remains uncertain. ^18^F-fluorodeoxyglucose PET (FDG-PET) imaging is a standard diagnostic tool for many cancers. However, its use in PABC staging is controversial due to potential radiation risks to the foetus.

**Methods:**

This retrospective case series analysed clinical data from six patients with high-risk PABC who underwent FDG-PET imaging for staging between 2022 and 2023. FDG-PET was based on locally implemented ultra-low-dose imaging protocols. The radiation doses to the foetus were dosimetrically estimated based on dose-per-unit activity values and correlated with postpartum neonatal outcomes.

**Results:**

The median foetal radiation dose was 0.975 mGy (range 0.6–1.5 mGy) and was below the threshold for deterministic toxicities. PET imaging upstaged nodal involvement in 33% of patients and influenced treatment decisions. FDG-PET imaging provided valuable staging information in all cases. No adverse foetal effects were observed.

**Conclusion:**

Ultra-low-dose FDG-PET imaging is a valuable tool providing accurate staging information to guide treatment decisions. The low radiation dose associated with this technique makes it a clinically acceptable modality for cancer staging in pregnant women. A larger case series is needed to precisely quantify foetal radiation doses and assess long-term safety.

## Introduction

Pregnancy-associated breast cancers (PABC) are defined as breast cancers diagnosed during pregnancy or the year following delivery and affect up to 1 in 3000 pregnancies worldwide [1]. In women under 30 years of age, as many as 20% of breast cancers are pregnancy-associated [2]. The overall incidence of PABC is expected to rise due to current trends in postponing pregnancy to later in life combined with the increased incidence of cancer in younger patients [3,4]. Managing PABC is particularly challenging due to the need to balance effective cancer treatment with the safety of both the foetus and the mother. This requires a multidisciplinary approach involving diagnostic, oncological, and obstetric expertise to ensure comprehensive care. Physiological changes, such as breast enlargement, changes in breast tissue, and nipple discharge, can mask the signs of breast cancer [4]. This can delay the diagnosis, leading to presentations with advanced disease resulting in poorer maternal outcomes and higher risk for preterm delivery, stillbirths, and neonatal mortality [3,5]. Moreover, cancer during pregnancy is known to be associated with major adverse outcomes, including premature rupture of membranes, preeclampsia, obstetric haemorrhage, a seven-fold risk of venous thromboembolism, and a 42-fold risk of maternal death [6].

Recent therapeutic advances and changes in the management of PABC have improved maternal outcomes while reducing the risk of toxicity to the foetus [1,7,8]. Despite the unique challenges posed by pregnancy, national and international guidelines advocate for parity in the treatment of breast cancer between pregnant and nonpregnant women [9,10]. There is broad agreement on the importance of starting definitive treatment early in PABC. Accurate staging before initiating systemic therapy is essential, particularly for high-risk patients. However, current guidelines remain vague regarding the type of imaging that should be offered in pregnancy. Furthermore, physicians are often concerned about the perceived risks to the foetus from maternal exposure to radiation.

In most cases, staging of PABC relies on a combination of ultrasound and mammography [6]. However, the use of advanced imaging techniques such as computed tomography (CT), PET, and MRI remains controversial in pregnant patients, in contrast to the more comprehensive staging approach typically used for nonpregnant patients. The uncertainty stems from concerns about foetal exposure to ionising radiation from X-ray, CT, and PET-CT imaging and the potential long-term effects. Breast MRI is typically avoided during pregnancy due to concerns about the potential long-term effects of gadolinium on the foetus, which remain unclear [11–13]. In nonpregnant patients with breast cancer, radiological staging is indicated in cases with high-risk characteristics such as large tumour size (≥50 mm), involved axillary lymph nodes, aggressive biology [high grade or proliferation index, triple-negative disease, human epidermal growth factor receptor 2 (HER2)-positive disease], or where the clinical picture is suggestive of distant metastatic disease [13,14].

More recently, national and international guidelines have supported the use of 18F-fluorodeoxyglucose PET (FDG-PET) for both initial staging and treatment response assessment in breast cancer. This imaging modality has shown significant advantages over traditional methods, offering more detailed and accurate information about disease spread and treatment efficacy, which aids in optimising patient management [14–17].

FDG-PET imaging delivers a radiation dose that is directly proportional to the amount of FDG tracer administered. In PET-CT scans, an additional radiation dose comes from the low-dose CT component used for anatomical reference. However, this additional radiation dose is avoided when using hybrid digital positron emission tomography-magnetic resonance (PET-MR) scanners, which use MRI sequences for localisation and attenuation correction, eliminating the need for CT [18]. There is an increasing body of evidence demonstrating that the overall foetal radiation exposure with FDG-PET imaging, performed on contemporary scanners, is below the threshold dose for deterministic effects and at very low risk for stochastic effects [19–23]. This is especially true when using dedicated ultra-low-dose FDG-PET imaging protocols. The radiation dose on many newer digital PET scanners is under 1.5 mGy [20–26], lower than the radiation doses of 15–50 mGy to FDG-PET often mentioned in guidelines [27] and significantly lower than the threshold dose for deterministic effects of 100 mGy [28,29].

Despite increasing safety data, many clinical guidelines remain cautious about the use of FDG-PET imaging in PABC based on outdated information and overestimated radiation risks. Current evidence suggests that FDG-PET can significantly impact the clinical management of up to one-third of patients with pregnancy-associated cancers by providing crucial staging information that can influence treatment decisions [24]. This discrepancy highlights the need for the more informed use of FDG-PET in these complex cases.

In this case series, we discuss the safety and clinical impact of low-dose PET imaging in PABC based on our experience in a major tertiary breast cancer referral centre in the UK.

## Materials and methods

This is a retrospective analysis of routinely collected clinical data, conforming to an observational, noncomparative, single-centre case series report. Clinical data were retrieved from the local electronic patient database and anonymised for further analysis with the purpose of standard-of-care service evaluation. According to the National Institute for Health and Care Research in the UK, retrospective data analysis for service evaluation is absolved from ethics committee approval. As part of standard local clinical practice, all patients provided informed consent by signing a consent form before PET imaging and agreed to anonymously share their data and imaging results as part of this case series.

### Patients

This analysis included consecutive patients with histologically confirmed high-risk PABC referred to FDG-PET imaging for cancer staging due to high-risk features, including the suspicion of locally advanced or metastatic disease. All patients were under the care of the maternal medicine multidisciplinary team (MDT) and were closely monitored by a specialist team throughout the duration of their pregnancy and in the postpartum period. The clinical need for cancer staging, referral for FDG-PET imaging, and therapy management was discussed, agreed upon, and documented in a breast cancer MDT meeting. Patients and children remained further under the care of specialist teams (medical oncology, maternal medicine, and paediatrics, respectively), with complex clinical decisions being re-discussed and agreed at MDT meetings.

### Imaging protocol

Imaging has been performed on a 3 Tesla PET-MR scanner (Biograph mMR, Siemens Healthineers) using standard MR attenuation correction sequences with a scan time of 7 min/bed position [30].

In case of claustrophobic or MR-incompatible patients, scanning was performed on a high-definition digital PET-CT scanner (Siemens Biograph Vision 600, Siemens Healthineers) associated with an ultra-low-dose CT protocol (35 mAs, 100 kV, cut: 5 mm, pitch: 0.8). All PET and MR images were acquired from vertex to mid-thighs, with the exception of the ultra-low-dose CT, which was performed from vertex to the lower part of the kidneys, excluding the foetus from the field-of-view. PET imaging was carried out at the University College London Hospitals (UCLH) site only.

No consensus exists on the recommended protocol for women with a pregnancy-associated cancer requiring FDG-PET imaging. For FDG-PET imaging, the standard protocol dosage is expected to be between 3.7 and 5.9 MBq/kg body weight as outlined in the European Association of Nuclear Medicine and Molecular Imaging and Society of Nuclear Medicine and Molecular Imaging guideline [31]. We have implemented a low-dose FDG-PET imaging protocol for pregnant women imaging (1.5 MBq/kg body weight ± 10%). This dedicated low-dose PET protocol results in 60–75% reduction in overall radiation dose compared to standard-of-care.

Besides the standard 4–6 hour fasting, patients have been encouraged to stay well-hydrated in preparation for PET imaging. Before FDG injection, patients have been asked to drink approximately 0.5–1 L of plain water. From 30 to 60 min after FDG administration (to scanning time) and up to 2.5 h after completion of imaging, patients have been advised to maintain a high-level of hydration and void urine frequently, to lower the radiation dose to the foetus that originates from the excreted FDG tracer accumulating into the maternal urinary bladder.

### Radiation dose

Foetal radiation dose from FDG was estimated using the conversion factors (mGy/MBq) from previously published dosimetric data in women in their second or third trimester of gestation who underwent FDG-PET imaging following an intense hydration & voiding protocol up to 3.5 h post-PET (second trimester, median 0.008 mGy/MBq; third trimester, 0.005 mGy/MBq) [32].

### Statistical analysis

The characteristics of the patients in this case series are summarised descriptively with categorical data presented as percentages and continuous data presented as median (range).

## Results

### Epidemiological characteristics of the study population

The patients’ demographic and clinical information are shown in Table 1.

**Table 1:** Demographic, histopathological, staging, and treatment details of patients investigated with FDG-PET imaging

Patients	Age	Gestational age (weeks)	Tumour histology	TNM by FDG-PET imaging	Treatment during pregnancy	Disease status after delivery	Treatment postpartum	Neonatal outcomes
Patient 1	42	28	G2 IDC ER3 PR8 HER2 negative (left breast) G2 IDC ER5 PR7 HER2 negative (left breast) G2 IDC ER6 PR8 HER2 negative (right breast)	T2N0M0 T1cN0M0 T4bN0M0	Declined by the patient	PD	NACT (EC-P)	Healthy
Patient 2	34	23	G2 IDC ER8 PR6 HER2 negative	T2N0M0	NACT (P)	PMR	Continued NACT (EC) WLE+SLNB Adjuvant endocrine therapy	Healthy
Patient 3	40	14	G3 IDC ER0 PR0 HER2 positive	T2N3M0 (AN-1, -2, IMN, SCF)	NACT (EC-P)	PMR	Continued NACT (TCHP) WLE+ANC Adjuvant TDM-1	Ventilatory support for 3 weeks Afterwards fully recovered
Patient 4	38	20	G2 IDC ER6 PR7 HER2 negative	T2N2M0 (AN-1)	NACT (EC-P)	PMR	WLE+SLNB Adjuvant endocrine therapy Abemaciclib	Healthy
Patient 5	39	31	G2 IDC ER0 PR0 HER2 negative	T1N0M0 (*Note*: BRCA 2 mutation – prior breast cancer treated with NACT)	NACT (P)	CMR	Continued NACT (PC) Mastectomy + SLNB	Healthy
Patient 6	38	29	G3 IDC ER6 PR5 HER2 negative	T4dN3M0 (AN-1, -2, -3)	NACT (EC)	PMR	Continued NACT (EC-P) Declined surgery Metastatic recurrence	Healthy

AN-1/2/3, level 1, 2 or 3 axillary lymph node(s); ANC, axillary node clearance; BRCA, breast cancer gene 2; CMR, complete metabolic response; EC, epirubicin/cyclophosphamide; ER, oestrogen receptor; G, tumour grade; HER2, human epidermal growth factor receptor 2; IDC, invasive ductal carcinoma; IMN, internal mammary lymph node(s); NACT, neoadjuvant chemotherapy; P, paclitaxel; PC, paclitaxel/carboplatin; PD, progressive disease; PMR, partial metabolic response; PR, Progesterone receptor; SCF, supraclavicular fossa lymph node(s); SLNB, sentinel lymph node biopsy; WLE, wide local excision; TCHP, docetaxel/carboplatin/trastuzumab/pertuzumab; TDM-1, trastuzumab/emtansine.

In this case series, six patients with PABC were referred for FDG-PET imaging by the Joint UCLH & WH Breast Cancer MDT, in discussion with the Maternal Medicine MDT, in the period between February 2022 and August 2023. The median maternal age was 38.5 (range 34–42) years. Four patients were diagnosed in the second trimester and two patients in the third trimester of pregnancy (Table 1).

Patients #1, #3, #4, and #6 presented with locally advanced breast cancer, which necessitated cancer staging, as per current standard practice.

Patient #2 and #5 did not have locally advanced disease, but were considered of high risk based on additional clinical information. Patient #2 had hormone receptor-positive, HER2-negative breast cancer. It is not conventional to administer neoadjuvant chemotherapy (NACT) in the disease subtype. However, breast surgery was deemed high risk due to prior pregnancy losses, and the MDT agreed to treat with NACT based on the results of a genomic risk assay (OncotypeDx) carried out on the core biopsy sample. Patient #5 had a BRCA2 mutation and had been treated curatively for breast cancer in the affected breast, 2 years before this presentation. The high-intermediate genomic risk results in patient #2 and presentation with recurrent breast cancer in patient #5 were grounds for recommendation of cancer staging by the MDT.

Systemic anticancer therapy in the form of NACT was offered in all cases and accepted by 83.3% (*n* = 5/6) of patients. NACT included chemotherapy with epirubicin/cyclophosphamide ± paclitaxel. One patient (patient #1) declined NACT while pregnant after discussing her options with the treating team (Table 1).

### Impact of FDG-PET on cancer staging

Cancer staging was completed with whole-body FDG-PET(-MR) in most patients (*n* = 5), while one patient opted for FDG-PET(-CT) due to claustrophobia. Ultra-low-dose FDG-PET image quality was deemed sufficient to inform the MDT in making clinical decisions in all cases. No metastatic disease was identified in any of the cases. PET imaging enabled an assessment of regional nodal involvement of level 2 and 3 axillary, internal mammary, and supraclavicular lymph node stations. All of these lymph node groups are not assessable by ultrasound.

**Fig. 1:**
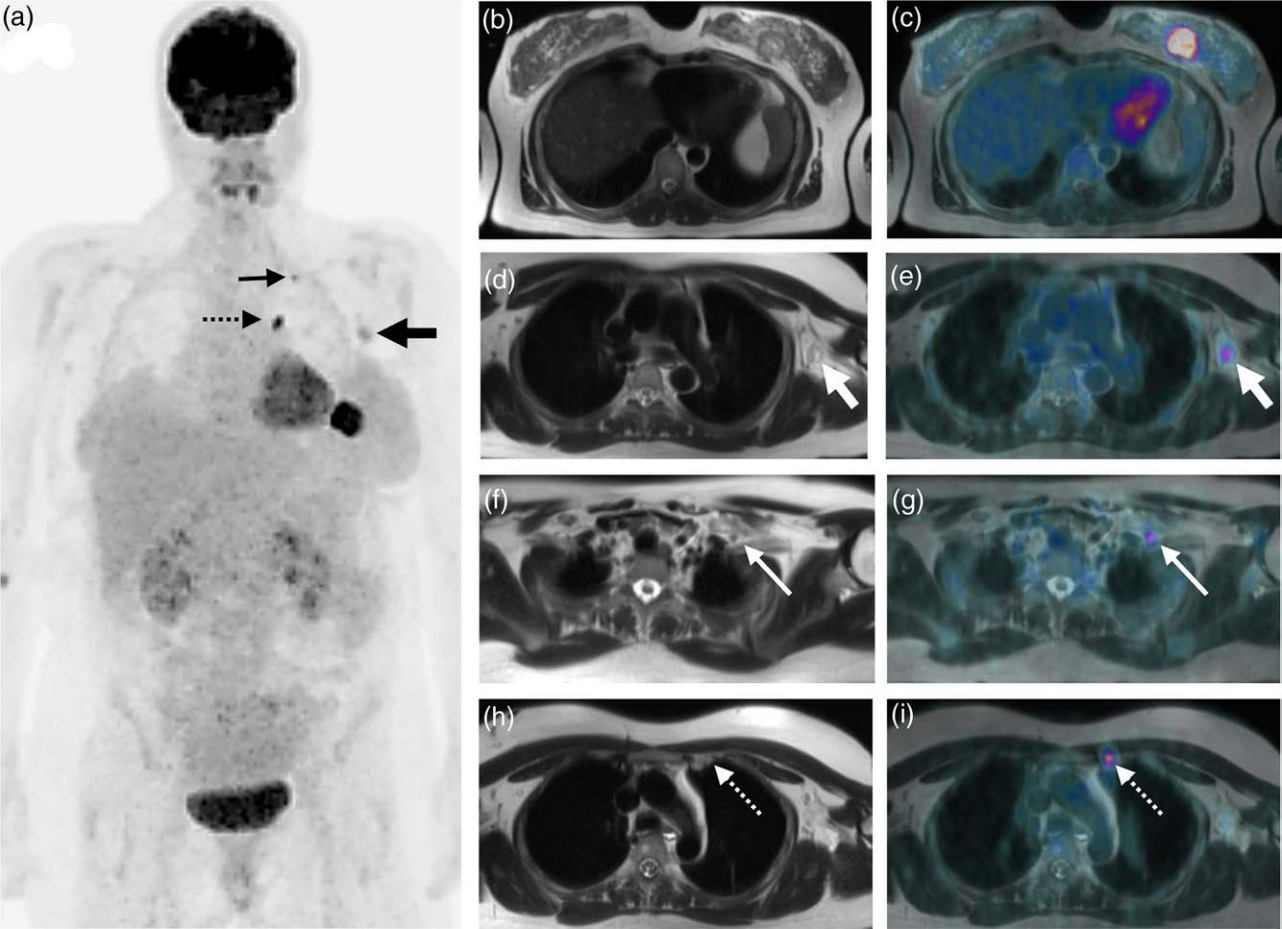
Patient #3. (a) Maximum intensity projection FDG-PET image depicting a left breast malignancy associated with an extensive locoregional nodal disease (arrows). T2-weighted MR images (b,d,f,h) and fused FDG-PET(-MR) images (c,e,g,i) show a large FDG-avid malignant process in the upper inner quadrant of the left breast (b,c). There are FDG-avid subcentimetre left axillary (thick arrow) (d,e), left supraclavicular fossa (thin arrow) (f,g), and left internal mammary chain (dotted arrow) (h,i) lymph node metastases, respectively.

FDG-PET imaging led to upstaging of nodal involvement in 33% of patients (*n* = 2/6). In both these patients, level 2 and level 3 regional lymph node involvement was identified on PET imaging. This information would not have been identified with conventional imaging modalities used in pregnancy, i.e., ultrasound imaging. Patient #3 was diagnosed with HER2 positive breast cancer (Fig. 1). Interval PET imaging carried out postpartum, following a set number of cycles of NACT (without HER2-directed therapy, as contraindicated in pregnancy), identified response in the nodal disease with no response in the primary tumour in the breast. The MDT considered this and elected to persevere with NACT in combination with the introduction of HER2-directed therapy in the postpartum period with a good clinical outcome to present.

### Maternal outcomes

NACT was tolerated well, with no grade 3 or above toxicities reported. None of the patients required NACT to be discontinued prematurely in an unplanned manner due to toxicities.

A caesarean section was performed for delivery in 83.3% (*n* = 5) of patients, and labour was induced in 16.7% (*n* = 1) of patients. The earliest delivery was at 34 weeks of gestation.

Breast surgery was performed postpartum in 83.3% (*n* = 5/6) of patients, including one patient who decided against proceeding with NACT during pregnancy (patient #1). One patient (patient #6) did not accept breast surgery postpartum and, unfortunately, subsequently relapsed with extensive metastatic disease.

### Radiation dose to the foetus

The dosimetric radiation dose estimates from FDG-PET to foetuses are summarised in Table 2.

**Table 2:** Dosimetry estimations of the patients investigated with FDG-PET imaging

Patients	Injected FDG-activity (MBq)	Imaging modality	Radiation dose to the foetus			Added risk of cancer in childhood (%)
			Low-dose PET (mGy)	Ultra-low-dose CT (mGy)	Total (mGy)	
Patient 1	128	PET-MR	0.652	–	0.652	0.01
Patient 2	112	PET-CT	0.952	0.6	1.552	0.01
Patient 3	131	PET-MR	1.113	–	1.113	0.01
Patient 4	134	PET-MR	1.139	–	1.139	0.01
Patient 5	196	PET-MR	0.999	–	0.999	0.01
Patient 6	153	PET-MR	0.780	–	0.780	0.01

MBq, megabequerel; mGy, miligray.

Overall, the radiation dose to foetuses was a median of 0.975 mGy. This ranged from a dose of 0.652 mGy with PET-MR and up to a dose of 1.552 mGy with PET-CT imaging (including the additional radiation dose of 0.6 mGy from ultra-low-dose CT), based on previously published dosimetric data [20,33]. Importantly, all the radiation levels were substantially lower than the threshold of 100 mGy, above which deterministic toxicities, such as miscarriage, foetal growth restriction, and malformations, are likely to occur [34]. Based on the available evidence and guidance, the risk of an adverse event, such as childhood cancer from a dose of 1 mGy, is 1 in 17,000, which translates into an estimated additional risk of 0.01% for childhood cancer [34] compared with a risk of 0.2% of cancer in general paediatric population [35], a 3–4% risk of congenital malformations, and a 15% chance for miscarriage in physiological pregnancies [33].

### Foetal outcomes

In this case series, one patient delivered a newborn at 34 weeks gestation who needed ventilatory support after birth, likely due to neonatal prematurity. The newborn was subsequently discharged from the hospital and is reported to be well. All other five patients delivered healthy newborns with normal Apgar scores (Table 1). On follow-up with the parents, no growth, developmental abnormalities, or childhood malignancies were reported at delivery for up to 24 months following delivery.

## Discussion

To our knowledge, this article is the first case series discussing the role of FDG-PET imaging in PABC in the UK. Herewith we report on ultra-low-dose FDG-PET imaging in cancer patients, mainly on breast cancer patients. We have demonstrated that in a limited number of cases, the use of ultra-low-dose FDG-PET imaging was not associated with any immediate or short-term consequences to the foetuses’ safety. Moreover, it provided more accurate staging before the use of NACT, which is utilised more frequently during the second and third trimester and helped tailor the management of the disease at surgery and postoperatively.

Patients with PABC represent a vulnerable patient population in which a careful multidisciplinary approach plays a key role in achieving optimal maternal and neonatal outcomes. A PABC diagnosis is often delayed due to physiological changes during pregnancy, leading to late presentations and poorer outcomes. Concerns about the use of standard-of-care imaging using ionising radiation and the associated harms to the unborn child can lead to delays in completing staging investigations and making treatment decisions with incomplete clinical information.

In this case series, we present six cases in which FDG-PET imaging permitted staging during the second or third trimesters of pregnancy with an ultra-low-dose PET protocol, limiting the exposure to ionising radiation. The case series highlights the clinical value of FDG-PET imaging in PABC in acquiring accurate and timely staging information. The use of FDG-PET imaging was effective in detecting regional nodal involvement and potential metastases. The extent of regional nodal involvement can be used to guide treatment decisions, especially adjuvant radiotherapy fields, i.e., inclusion of supraclavicular fossa (SCF) and internal mammary nodes. Nodal involvement was upstaged in 33% of cases with PET imaging. Serial FDG-PET imaging, which can be carried out in the postpartum period, provides the ability to monitor disease responses to treatment. This case series demonstrates the time-sensitive nature of staging information gained from FDG-PET imaging in pregnant patients. Based on the response to systemic treatment (as in patient three in this series), the extent of nodal disease may not be delineated if imaging is carried out after the commencement of systemic therapy, resulting in critical information being missed. Ultrasound of the neck and the regional lymph nodes, including the SCF, is not considered part of conventional staging in nonpregnant patients. In the absence of clinical suspicion, an ultrasound of the neck and SCF would not be considered in staging investigations, and small volume or deep-seated neck lymphadenopathy would have been missed without PET imaging.

Our dose estimations support the existing literature in proposing that FDG-PET can be offered to patients with a pregnancy-associated cancer diagnosis [15,36–40]. Other case studies and series support the application of this across pregnancy [41], however, our cases were limited to the second and third trimesters only. The foetal radiation dose is calculated by adding the amount of internal exposure from the tracer used for the PET scan to the external dose of CT. In this case series, we utilised FDG-PET(-MR) in most patients (83.3%, *n* = 5). As MRI does not involve ionising radiation, FDG-PET(-MRI) is a useful alternative to provide detailed imaging sequences without additional radiation. However, not all centres have the capability for FDG-PET(-MRI). If FDG-PET(-CT) must be considered, the total radiation dose to the foetus is below the recommended limits, particularly when applying a dedicated ultra-low-dose FDG protocol and using ultra-low-dose CTs [22,23,36].

The deterministic effects of radiation, including miscarriage, growth restriction, and malformations, can occur when the foetal radiation dose exceeds a threshold of 100 mGy [34]. On the other hand, stochastic effects, such as an increase in childhood malignancy risk, are not associated with a safety threshold [7]. Due to the potential risks to the foetus, whole-body CT or FDG-PET(-CT) scans are generally avoided in PABCs. These expose the foetus to radiation doses ranging from 10 to 50 mGy [1,8,42]. Diffusion-weighted, whole-body MRI (dwWB-MRI) has been suggested to be a feasible and clinically accurate alternative in PABC as it is not associated with radiation exposure [12]. However, the limited availability of dwWB-MRI, lack of standardised protocols for scanning and reporting, and limited published evidence make it difficult to draw impactful conclusions on its use.

While FDG-PET imaging performs well in detecting regional nodal involvement and distant metastases, calculating the foetal radiation dose from the FDG used for this purpose is an active field of research. There have been a series of case reports published in the literature on the use of FDG in pregnant women since 2008 [19–23,36,37]. Owing to recent technical improvements in PET imaging techniques, digital PET cameras allow imaging at ultra-low-dose FDG activities. When combined with an ultra-low-dose CT or even with exclusion of CT radiation with FDG-PET(-MR) imaging, the total radiation dose to the foetus is <1 mGy. This is 10- to 50-fold lower than the radiation estimates from standard FDG-PET(-CT)s, that is, 10–50 mGy as referred to in current guidelines [27].

The use of a reduced FDG dose protocol has, in our practice, allowed for satisfactory images to be obtained in all patients within this case series, supporting the role of FDG-PET in complementing the staging workup in pregnant women. Additional safety measures include advice for adequate hydration and regular bladder voiding to minimise prolonged tracer accumulation in close proximity to the uterus, although quantifying the benefits of this practice is challenging. FDG-PET imaging allowed the multidisciplinary team to make informed decisions, ensuring that treatment plans, such as NACT and adjuvant radiotherapy, were based on precise staging information.

Our recommendations for the use of FDG-PET in pregnancy for the purpose of cancer staging are as follows:

1. The clinical need and the decision to recommend cross-sectional imaging for cancer staging should be based on a joint MDT meeting recommendation, taking into consideration the mother’s benefit and risk to the foetus. It is advisable that all standard imaging modalities, including FDG-PET, are taken into discussion.
2. Delaying investigation until later in pregnancy should be considered only if it does not affect the mother’s risk and prognosis or limit her options for termination of pregnancy, should she wish to consider this option.
3. We recommend discussion with a nuclear medicine physician and medical physicist to gather information on protocols, dosimetry, and, most importantly, methods to limit radiation exposure as much as possible while still preserving a good quantitative accuracy and maintaining the diagnostic value of the scan.
4. A low-dose FDG-PET(-MR) imaging is advisable due to its low ionising radiation dose and absence of CT-related radiation.
5. If FDG-PET(-MR) is unavailable or if the patient cannot undergo MR due to safety reasons (e.g. metal implants), low-dose FDG-PET(-CT) imaging can be considered with inclusion of both low FDG-activity and an adapted low-dose or ultra-low dose CT protocol.
6. An individual radiation risk assessment should be performed based on the agreed protocol, and the potential dose to the foetus should be calculated. This information, alongside the information about the increase in cancer risk for the foetus and the general childhood cancer risk, should be saved in the patient’s record. The procedure and the risks are then discussed with the patient. Women should be counselled that FDG does accumulate in the foetus, but the benefits of the information obtained are likely to outweigh any potential risks to the foetus.
7. If the patient wishes to proceed, informed consent should be obtained on the day of the procedure, allowing the patient time to consider the risks and ask any questions.
8. In addition to the low FDG-activity administered, ensuring adequate hydration to the patient is important. This can be achieved by using oral and/or IV fluids judiciously while being mindful to avoid volume overload. It is advisable for women to void urine before image acquisition to prevent prolonged dwell time of the tracer in the bladder, given its proximity to the uterus. The use of diuretics or catheterisation to minimise foetal exposure to the tracer may also be considered.
9. The clinical follow-up of the patient and child is strongly recommended. This would include monitoring for maternal cancer-related outcomes, pregnancy-related outcomes (e.g. live birth, medical termination of pregnancy, or abortion and corresponding gestation age, neonate’s status at birth and postpartum), child’s development (e.g. malformation, mental retardation), and the occurrence of childhood cancer.

To conclude, FDG-PET imaging represents a pivotal tool in the optimal management of PABC, ensuring better outcomes through timely interventions and enhanced treatment planning. Providing accurate information about staging also has a positive impact on the patients’ experience by reducing anxiety, as a diagnosis of PABC is often delayed due to the associated physiological changes in the breast and uncertainty as patients progress through the diagnostic pathway. Further work is needed to quantify the foetal doses from FDG-PET imaging in patients with PABC in a larger case series.

## Acknowledgements

We acknowledge the help of the UCLH PET-MRI (James Vincent), the PET-CT scanner unit (Aimee Pearson), and the Institute of Nuclear Medicine (Rayjanah Allie) for their support in planning and performing the scans.

K.D. and S.V. have conceptualised the analysis. K.D. and P.F. collected and analysed the clinical data. K.D., P.F., E.S., E.P., F.R., R.R., D.O., J.F., D.B., M.B., I.A., M.C., N.P., and S.H. have reviewed the clinical data. Y.K., D.W., C.S., M.R., M.A., J.B., and S.V. have reviewed the nuclear medicine data. K.D., P.F., and S.V. wrote the first draft, which has been revised by all authors. All authors have read, edited, reviewed, amended, and authorised the paper.

All authors consented for publication.

Data is available on request from the authors, in conformity to national clinical data governance on patient privacy and ethical considerations.

### Conflicts of interest

There are no conflicts of interest.
